# Safe discharge for patients admitted for lower gastrointestinal bleeding (LGITB): derivation and validation of a novel scoring system

**DOI:** 10.1186/s12876-023-02950-w

**Published:** 2023-10-09

**Authors:** Yue Zhao, Madeline Yen Min Chee, Rehena Sultana, Winson Jianhong Tan

**Affiliations:** 1 Ministry of Health Holdings, 110 Sengkang E Way, Singapore, 544886 Singapore; 2 Ministry of Health Holdings, Singapore, Singapore; 3 Duke-NUS Medical School, Singapore, Singapore; 4 Department of General Surgery, Sengkang General Hospital, Singapore, Singapore

**Keywords:** Gastrointestinal bleeding, Emergency surgery

## Abstract

**Aim:**

Bleeding from the lower gastrointestinal tract (LGITB) is a common clinical presentation. Recent guidelines have recommended for incorporation of clinical risk assessment tools in the management for LGITB. We derived and validated a novel clinical scoring system to predict safe discharge after LGITB admission, and compared it to other published scoring systems in current literature.

**Methods:**

A retrospective cohort of 798 patients with LGITB from August 2018 to March 2021 was included in the derivation cohort. Multivariate binary logistic regression was performed to identify significant clinical variables predictive of safe discharge. A clinical scoring system was developed based on the results, and validated on a prospective cohort of 312 consecutive patients with LGITB from April 2021 to March 2022. The performance of the novel scoring system was compared to other LGITB clinical risk assessment scores via area under the receiver operating characteristics curve (AUROC) analysis.

**Results:**

Variables predictive of safe discharge included the following; absence of previous LGITB admission, absence of ischemic heart disease, absence of blood on digital rectal examination, absence of dizziness or syncope at presentation and the systolic blood pressure and haemoglobin levels at presentation. The novel score had an AUROC of 0.907. A cut-off point of 4 provided a sensitivity of 41.9%, specificity of 97.5%, positive predictive value of 96.4% and negative predictive value of 51.5% for prediction of safe discharge. The score performs comparably to the Oakland score.

**Conclusion:**

The novel LGITB clinical risk score has good predictive performance for safe discharge in patients admitted for LGITB.

## Introduction

Bleeding from the gastrointestinal tract (GITB) can be divided based the relation of the source with the ligament of Treitz. Upper GITB (UGITB) is defined as haemorrhage proximal to the ligament of Treitz while lower GITB (LGITB) is defined as haemorrhage beyond this anatomical landmark. GITB is a common reason for hospital admissions, with an estimated 1.74 events per 1000 individuals annually [1]. LGITB has been estimated to account for at least 20% of all GITB [2, 3] and has been on an increasing trend recently. Between 1996 to 2005, the incidence of LGITB has nearly doubled from 20 per 100, 000 individuals to 33 per 100, 000 individuals [4].

The clinical outcomes of LGITB can be highly variable. While most patients with LGITB have spontaneous clinical improvement and does not require hospitalisation [5, 6], it has been estimated that nearly 30% of them will require either transfusion of blood products or urgent interventions in the form of endoscopy, interventional radiology, or surgery [7]. As such, accurate stratification of LGITB patients is important to ensure safe management and optimal utilization of scarce healthcare resources. Indeed, the 2019 British Society of Gastroenterology (BSG) guidelines on the diagnosis and management of acute LGITB has recommended the incorporation of risk assessment tools in the management algorithm for LGITB [8].

Multiple clinical scores have been published to assess the outcomes in patients who presented with LGITB. However, the outcomes measured are heterogeneous and most of the scores have not been validated in an external population [7, 9–16]. This study aims to develop a scoring system to predict safe discharge in patients with LGITB. The derived scoring system is validated in a prospective cohort and its performance compared against other risk assessment models in current literature. The LGITB risk assessment models included for comparison are the Oakland score and the Glasgow-Blatchford score (GBS) [16].

## Material and methods

The study comprises a derivation cohort and a separate validation cohort. The derivation cohort was derived from patients aged ≥ 21 years who were admitted to the surgical unit in Sengkang General Hospital between August 2018 and March 2021 with a diagnosis of LGITB. Eligible patients were identified from the hospital database by key terms available within the discharge diagnosis, including 'gastrointestinal bleeding', 'lower gastrointestinal bleeding', 'rectal bleeding', 'intestinal bleeding', 'diverticular bleed', 'colorectal cancer', 'colitis', 'haemorrhoids'. Exclusion criteria included patients who had UGITB (as defined by endoscopic diagnosis of a bleeding lesion proximal to the Ligament of Treitz), elective admissions via the clinic or patients who had opted for palliative care and comfort measures upon admission due to other comorbidities. The validation cohort was a prospective cohort of consecutive patients who were admitted to our unit between April 2021 to March 2022, with similar inclusion and exclusion criteria applied.

Our definition of safe discharge was modified from Oakland [16]. This was a composite outcome, determined by the absence of all of the events following admission: transfusion of red blood cells, therapeutic endoscopic procedures (e.g. adrenaline injection, endoscopic clipping), therapeutic interventional radiological procedures (e.g. mesenteric embolization), surgical procedures for haemostasis; rebleeding (defined as a reduction in haematocrit concentration of 20% or more after 24 h of stability) [16]; in-hospital or 28-day mortality (all causes); and 28-day readmission due to LGITB. The study was approved by the local Institutional Review Board.

Data collected from the eligible patients included their demographic information, past medical history, Charlson score, chronic medications, symptoms on admission, vital signs at the Emergency Department triage, laboratory results on admission, endoscopic findings (if performed), interventions performed, high dependency (HD) or intensive care unit (ICU) admission, in-hospital deaths and readmissions or deaths within 28 days related to LGITB. The derivative phase focused on the identification of significant variables predictive of safe discharge. Categorical variables were analysed using the Pearson X2 test, and continuous variables were analysed with the Kruskal–Wallis test. Variables which were identified to be statistically significant in the prediction of safe discharge for LGITB (defined as a *p*-value ≤ 0.1) were then entered into a multivariate binary logistic regression model. Variables found to be significant on the multivariate model (defined as p ≤ 0.05) were included into the clinical score. Each variable was assigned points corresponding to its unstandardized coefficient on the multivariate model to develop the scoring system. The scoring system was validated by applying the score on the validation cohort. The performance of the model was assessed with calculation of the area under the ROC curve (AUROC), and subsequently compared to the AUROCs of other published clinical scores using the method described by DeLong et al. [17].

Statistical analysis was performed using IBM SPSS Statistics for Windows, Version 26.0. (Armonk, NY: IBM Corp) for the derivation and validation of the scoring system. Comparison of the AUROC curves was performed using MedCalc Statistical Software version 19.2.6 (MedCalc Software Ltd, Ostend, Belgium; https://www.medcalc.org; 2020).

## Results

**Fig. 1:**
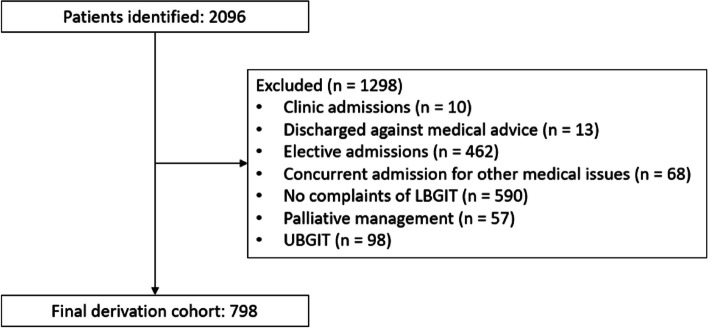
Flowchart of patients recruited for derivation

There were a total of 2096 patients who were screened for inclusion into the derivation cohort. 1298 patients were excluded due to admission from clinic instead of ED (10), discharged against medical advice before treatment was completed (13), elective admissions for diagnostic or therapeutic purposes (462), absence of LGITB symptoms (590), admission for other concurrent medical issues (68), palliative management opted by patient or family on admission (57), and presence of UGITB (98). The final derivative cohort had 798 patients (Fig. 1). The validation cohort had 312 patients.

Comparison of the demographic characteristics of the derivation and validation cohorts is illustrated in Table 1. There were no significant differences between the two cohorts with regards to median age, previous LGITB admissions, median Charlson score, hypertension, diabetes, ischemic heart disease, chronic kidney disease, chronic liver disease, usage of antiplatelets, usage of anticoagulants, presence of diarrhoea, presence of syncope and/or dizziness, presence of abdominal tenderness, digital rectal examination (DRE) findings of blood, median systolic blood pressure, median diastolic blood pressure, median heart rate, median total white count, median platelet counts, median urea and median creatinine levels. However, the validation cohort had significantly more females (51.6% vs 42.0%, *p* = 0.004), lower initial median Hb levels (11.5 vs 11.9, *p* = 0.048), higher median INR (1.0 vs 0.9, *p* < 0.001), lower median APTT (25.9 vs 26.8, *p* < 0.001) and lower median bicarbonate levels (22.1 vs 22.4, *p* = 0.017) (Table 1). In terms of aetiology of LGITB, the validation cohort had a higher proportion of colitis (18.3% vs 12.2%, *p* = 0.008). Otherwise, the cohorts were similar in terms of the distribution of diverticular disease (33.8% vs 34.0%, *p* = 0.965), malignancy (8.0% vs 5.6%, *p* = 0.144) and haemorrhoids (33.3% vs 39.6%, *p* = 0.053) as the aetiology of the LGITB. Both cohorts were also similar in the proportion of patients who were deemed to have met the criteria for safe discharge (62.5% vs 61.2%, *p* = 0.685) (Table 1). 643 patients underwent colonoscopic evaluation (57.9%), while 360 patients underwent gastroscopic evaluation (32.4%). There was no significant difference in the median length of stay between both cohorts (*p* = 0.299). There were 2 mortalities while inpatient (0.18%), and 7 deaths within 28 days of discharge (0.63%).

**Table 1:** Comparison of baseline characteristics of derivative and validation cohorts

**Variable**	**Derivative (** ***n*** ** = 798)**	**Validation (** ***n*** ** = 312)**	***P*** **-value**
**Clinical demographics**			
Gender (*n* = 1110)			
Female	335 (42.0%)	161 (51.6%)	**0.004**
Male	463 (58.0%)	151 (48.4%)	
Age (*n* = 1110)	66 (IQR 54–77)	69 (IQR 54–79)	**0.08**
**Medical history and medications**			
Previous LGITB admission (*n* = 1110)	131 (16.4%)	57 (18.3%)	0.459
Presence of hypertension (*n* = 1110)	381 (47.7%)	136 (43.6%)	0.212
Presence of diabetes (*n* = 1110)	619 (77.6%)	241 (77.2%)	0.907
Presence of ischemic heart disease (*n* = 1110)	157 (19.7%)	57 (18.3%)	0.594
Presence of chronic kidney disease (*n* = 1110)	88 (11.0%)	33 (10.6%)	0.829
Presence of liver disease (*n* = 1110)	12 (1.5%)	6 (1.9%)	0.619
Charlson Score (*n* = 1110)	3 (IQR 1–4)	3 (IQR 1–5)	0.129
Usage of antiplatelets (*n* = 1110)	212 (26.6%)	80 (25.6%)	0.753
Usage of anticoagulation (*n* = 1110)	46 (5.8%)	24 (7.7%)	0.235
**Signs and symptoms at presentation**			
DRE findings of blood (*n* = 1093)	563 (71.8%)	238 (77.0%)	**0.08**
Presence of diarrhea (*n* = 1108)	108 (13.6%)	48 (15.4%)	0.434
Presence of abdominal tenderness (*n* = 1110)	97 (12.2%)	35 (11.2%)	0.664
Presence of syncope or dizziness (*n* = 1052)	219 (28.9%)	87 (29.7%)	0.788
**Vital signs and laboratory results at presentation**			
SBP at triage (*n* = 1109)	130 (IQR 115–149)	131.0 (IQR 117–151.8)	0.415
DBP at triage (*n* = 1109)	76 (IQR 69–85)	77 (IQR 68.3–84.8)	0.904
HR at triage (*n* = 1109)	83 (IQR 73–95)	85 (IQR 75–97)	0.068
Initial Hb (*n* = 1110)	11.9 (IQR 9.9–13.4)	11.5 (IQR 9.13–13.3)	**0.048**
TW count (*n* = 1110)	8.28 (IQR 6.45–10.7)	8.39 (IQR 6.83–10.8)	0.428
Platelet count (*n* = 1110)	254 (IQR 205–313)	262 (IQR 211–315)	0.270
INR levels (*n* = 1011)	0.97 (IQR 0.94–1.02)	1.0 (IQR 0.97–1.04)	** < 0.001**
APTT levels (*n* = 979)	26.8 (IQR 24.9–28.8)	25.9 (IQR 23.6–27.9)	** < 0.001**
Urea levels (*n* = 1107)	5.30 (IQR 4.10–7.30)	5.55 (IQR 4.10–7.65)	0.209
Bicarbonate levels (*n* = 1107)	22.4 (IQR 20.8–24.0)	22.1 (IQR 20.1–23.7)	**0.017**
Creatinine levels (*n* = 1108)	77.0 (IQR 60.0–99.5)	76.0 (IQR 62.0–94.0)	0.793
Aetiology of LGITB (*n* = 1110)			
Colitis (*n* = 154)	97 (12.2%)	57 (18.3%)	**0.008**
Diverticular disease (*n* = 376)	270 (33.8%)	106 (34.0%)	0.965
Haemorrhoids (*n* = 420)	316 (39.6%)	104 (33.3%)	0.053
Malignancy (*n* = 70)	45 (5.6%)	25 (8.0%)	0.144
Length of stay (*n* = 1110)	3.0 (IQR 2.0–4.0)	3.0 (IQR 2.0–4.0)	0.299
Safe discharge (*n* = 1100)	499 (62.5%)	191 (61.2%)	0.685

Data represented in numbers (%), mean (standard deviation) or median (inter-quartile range)

*DRE* Digital rectal examination, *SBP* Systolic blood pressure, *DBP* Diastolic blood pressure, *HR* Heart rate, *Hb* Haemoglobin, *TW* Total whites, *INR* International Normalised Ratio, *APTT* Activated partial thromboplastin time

In the derivation cohort, 19 clinical variables were significantly associated with safe discharge on univariate analysis: female gender, prior LGITB admission, absence of diabetes, absence of ischemic heart disease, absence of liver disease, no antiplatelet usage, absence of DRE findings of blood, presence of abdominal tenderness, absence of syncope or dizziness, lower Charlson score, higher SBP and DBP at triage, lower HR at triage, higher initial Hb levels, lower platelet count, lower INR levels, higher APTT levels, lower urea and creatinine levels, and higher bicarbonate levels (Table 2). 139 cases (17.4%) were excluded from the multivariate analysis due to missing data. Charlson score was not entered into the multivariate analysis due to concerns of collinearity.

**Table 2:** Univariate analysis of variables associated with safe discharge

**Variable**	**Safe discharge (** ***n*** ** = 499)**	**Not safe for discharge (** ***n*** ** = 299)**	***P*** **-value**
**Clinical demographics**			
Gender (*n* = 1110)			
Female	226 (45.3%)	109 (36.5%)	**0.014**
Male	273 (54.7%)	190 (63.5%)	
Age (*n* = 798)	66 (IQR 52–76)	66 (IQR 52–76)	0.135
**Medical history and medications**			
Previous LGITB admission (*n* = 798)	441 (88.4%)	226 (75.6%)	** < 0.001**
Presence of hypertension (*n* = 798)	251 (50.3%)	166 (55.5%)	0.153
Presence of diabetes (*n* = 798)	94 (18.8%)	85 (28.4%)	**0.002**
Presence of ischemic heart disease (*n* = 798)	79 (15.8%)	78 (26.1%)	** < 0.001**
Presence of chronic kidney disease (*n* = 798)	56 (11.2%)	32 (10.7%)	0.820
Presence of liver disease (*n* = 798)	4 (0.8%)	8 (2.7%)	**0.066**
Charlson Score (*n* = 798)	3 (IQR 1–4)	4 (IQR 2–5)	** < 0.001**
Usage of antiplatelets (*n* = 798)	120 (24.0%)	92 (30.8%)	**0.037**
Usage of anticoagulation (*n* = 798)	25 (5.0%)	21 (7.0%)	0.238
**Signs and symptoms at presentation**			
DRE findings of blood (*n* = 784)	315 (64.2%)	248 (84.6%)	** < 0.001**
Presence of diarrhea (*n* = 796)	73 (14.7%)	35 (11.7%)	0.245
Presence of abdominal tenderness (*n* = 798)	77 (15.4%)	20 (6.7%)	** < 0.001**
Presence of syncope or dizziness (*n* = 759)	80 (16.9%)	139 (48.4%)	** < 0.001**
Days of symptoms (*n* = 769)	1 (IQR 1–3)	1 (IQR 1–3)	0.839
**Vital signs and laboratory results at presentation**			
SBP at triage (*n* = 797)	135.5 (IQR 120–154)	121.0 (IQR 108–137)	** < 0.001**
DBP at triage (*n* = 797)	79 (IQR 70–87)	72 (IQR 65–79)	** < 0.001**
HR at triage (*n* = 797)	81 (IQR 71–91)	88 (IQR 76–99)	** < 0.001**
Initial Hb (*n* = 798)	12.8 (IQR 11.4–14.1)	9.3 (IQR 7.3–11.5)	** < 0.001**
TW count (*n* = 798)	8.07 (IQR 6.38–10.46)	8.50 (IQR 6.50–11.1)	0.351
Platelet count (*n* = 798)	249 (IQR 205–298)	263 (IQR 204–338)	**0.029**
INR levels (*n* = 724)	0.96 (IQR 0.94–1.0)	0.98 (IQR 0.95–1.03)	** < 0.001**
APTT levels (*n* = 706)	27.3 (IQR 25.7–29.2)	25.6 (IQR 23.2–28.2)	** < 0.001**
Urea levels (*n* = 797)	5.10 (IQR 3.90–6.80)	5.90 (IQR 4.40–8.10)	** < 0.001**
Bicarbonate levels (*n* = 797)	22.5 (IQR 21.1–24.4)	22.2 (IQR 20.4–23.6)	**0.002**
Creatinine levels (*n* = 797)	74.0 (IQR 59–96)	83.0 (IQR 64.0–103.0)	** < 0.001**

Data represented in numbers (%), mean (standard deviation) or median (inter-quartile range)

*DRE* Digital rectal examination, *SBP* Systolic blood pressure, *DBP* Diastolic blood pressure, *HR* Heart rate, *Hb* Haemoglobin, *TW* Total whites, *INR* International Normalised Ratio, *APTT* Activated partial thromboplastin time

In multivariate binary logistic regression, the significant variables were previous LGITB admission (OR 1.93, 95% CI 1.11–3.35), presence of ischemic heart disease (OR 2.46, 95% CI 1.19–5.09), DRE findings of blood (OR 3.51, 95% CI 1.95–6.29), presence of syncope or dizziness (OR 2.21, 95% CI 1.35–3.63), lower SBP (OR 0.983, 95% CI 0.97–0.995) and lower initial Hb levels (OR 0.55, 95% CI 0.489–0.619) (Table 3).

**Table 3:** Multivariate binary logistic regression model to predict for safe discharge

**Variables**	**Odds ratio (95% CI)**	***P*** **-value**	**Unstandardised coefficient**
Female gender	1.53 (0.956–2.434)	0.077	0.422
**No prior LGITB admission**	**1.93 (1.11–3.35)**	**0.02**	**0.657**
No history of DM	1.18 (0.688–2.03)	0.543	0.168
**No history of IHD**	**2.46 (1.19–5.09)**	**0.016**	**0.899**
No history of chronic liver disease	2.75 (0.538–14.1)	0.224	1.012
No usage of antiplatelets	1.53 (0.791–2.95)	0.207	0.424
**Blood absent on DRE**	**3.51 (1.95–6.29)**	** < 0.001**	**1.25**
Presence of abdominal tenderness	1.70 (0.759–3.81)	0.197	0.532
**Absence of syncope or dizziness**	**2.21 (1.35–3.63)**	**0.002**	**0.794**
**SBP at triage**	**1.02 (1.01–1.03)**	**0.007**	**0.18**
DBP at triage	0.983 (0.959–1.01)	0.167	- 0.017
HR at triage	0.991 (0.976–1.01)	0.227	- 0.009
**Initial Hb count**	**1.78 (1.62–2.04)**	** < 0.001**	**0.597**
Platelet count	1 (0.997–1.00)	0.732	0.00
INR level	0.958 (0.954–1.47)	0.845	- 0.043
APTT level	1.02 (0.969–1.08)	0.426	0.022
Urea level	1.00 (0.923–1.09)	0.976	0.001
Bicarbonate level	1.02 (0.928–1.12)	0.715	0.017
Creatinine level	0.999 (0.996–1.00)	0.481	- 0.001

SBP readings and initial Hb levels were then categorised according to appropriate categories based on their unstandardised coefficient. The multivariate binary logistic regression model was repeated with the same 19 clinical variables, but with SBP and Hb analysed as categorical variables. In the second regression model, the categories for SBP were > 135 mmHg, 120-134 mmHg, 105-119 mmHg, 90-104 mmHg and < 90 mmHg; the categories for initial Hb levels were > 13 g/dL, 12–12.9 g/dL, 11–11.9 g/dL, 10–10.9 g/dL and < 10 g/dL. Adjustments to these categories of SBP and Hb were required as the unstandardised coefficients for certain categories were similar. Thus, the SBP categories of 105-119 mmHg and 120-134 mmHg were combined, and the initial Hb categories of 11–11.9 g/dL and 12–12.9 g/dL were combined. In the final regression model, the categories for SBP were > 135 mmHg, 120-134 mmHg, 90-119 mmHg and < 90 mmHg; whilst the categories for initial Hb readings were > 13 g/dL, 11–12.9 g/dL, 10–10.9 g/dL and < 10 g/dL (Table 4).

**Table 4:** Multivariate binary logistic regression model with modified Hb and SBP categories to predict for safe discharge

**Variables**	**Odds ratio (95% CI)**	***P*** **-value**	**Unstandardised coefficient**
Female gender	1.38 (0.866–2.19)	0.176	0.320
**No previous LGITB admission**	**2.11 (1.21–3.69)**	**0.008**	**0.749**
No DM	1.17 (0.679–2.0)	0.580	0.152
**No IHD**	**2.59 (1.24–5.40)**	**0.011**	**0.951**
No chronic liver disease	2.83 (0.531–15.1)	0.223	1.04
No usage of antiplatelets	1.57 (0.801–3.07)	0.190	0.449
**DRE findings of no blood**	**3.31 (1.88–5.85)**	** < 0.001**	**1.20**
Presence of abdominal tenderness	1.87 (0.850–4.10)	0.120	0.624
**Absence of syncope or dizziness**	**2.51 (1.55–4.08)**	** < 0.001**	**0.922**
**SBP at triage (reference: SBP ≥ 135 mmHg)**			
**SB** ***P*** ** < 90 mmHg**	**0.0562 (0.00773–0.409)**	**0.004**	**-2.88**
**SBP 90-119 mmHg**	**0.410 (0.219–0.768)**	**0.005**	**- 0.891**
**SBP 120-134 mmHg**	**0.540 (0.307–0.952)**	**0.033**	**- 0.616**
DBP at triage	0.986 (0.964–1.01)	0.233	- 0.014
HR at triage	0.988 (0.974–1.00)	0.098	- 0.012
**Initial Hb count (reference: Hb ≥ 13.0 g/dL)**			
**Hb < 10 g/dL**	**0.0328 (0.0163–0.0661)**	** < 0.001**	**- 3.418**
**Hb 10–11.9 g/dL**	**0.209 (0.0978–0.447)**	** < 0.001**	**- 1.57**
**Hb 12–12.9 g/dL**	**0.346 (0.192–0.622)**	** < 0.001**	**- 1.06**
Platelet count	1.0 (0.997–1.00)	0.828	0.00
INR level	0.920 (0.621–1.36)	0.66	- 0.084
APTT level	1.04 (0.981–1.09)	0.237	0.035
Urea level	0.994 (0.918–1.09)	0.908	- 0.006
Bicarbonate level	1.01 (0.924–1.11)	0.731	0.014
Creatinine level	0.999 (0.996–1.00)	0.475	- 0.001

The significant variables based on the final regression model were previous LGITB admission (OR 2.11, 95% CI 1.21–3.69), presence of ischemic heart disease (OR 2.59, 95% CI 1.24–5.40), DRE findings of blood (OR 3.31, 95% CI 1.99–5.86), presence of syncope or dizziness (OR 2.51, 95% CI 1.55–4.09), SBP 120-134 mmHg (OR 1.85, 95% CI 1.05–3.26), 90-119 mmHg (OR 2.44, 95% CI 1.30–4.56), < 90 mmHg (OR 17.8, 95% CI 2.45–129); initial Hb 11–12.9 g/dL (OR 2.89, 95% CI 1.61–5.21), 10–10.9 g/dL (OR 4.78, 95% CI 2.24–1.023) and < 10 g/dL (OR 30.5, 95% CI 15.1–61.5).

Development of the clinical score was based on the unstandardised coefficients of the clinical variables, rounded to the nearest 0.5 (Table 5). The final scoring system was derived as such: previous LGITB admission (1.5 points); history of IHD (2 points); DRE findings of blood (2.5 points); presence of syncope or dizziness (2 points); initial SBP > 135 mmHg (0 point), 120-134 mmHg (1 point), 90-119 mmHg (2 points) and < 90 mmHg (6 points); initial Hb > 13 (0 point), 11–12.9 g/dL (2 points), 10–10.9 g/dL (3 points) and < 10 g/dL (7 points).

**Table 5:** Novel scoring system

**Variable**	**Score**
Previous LGITB admission	1.5
History of IHD	2
DRE findings of blood	2.5
Presence of syncope or dizziness	2
Initial SBP	
135 and above	0
120 to 134	1
90 to 119	2
Below 90	6
Initial haemoglobin	
13 and above	0
11 to 12.9	2
10 to 10.9	3
Less than 10	7

**Fig. 2:**
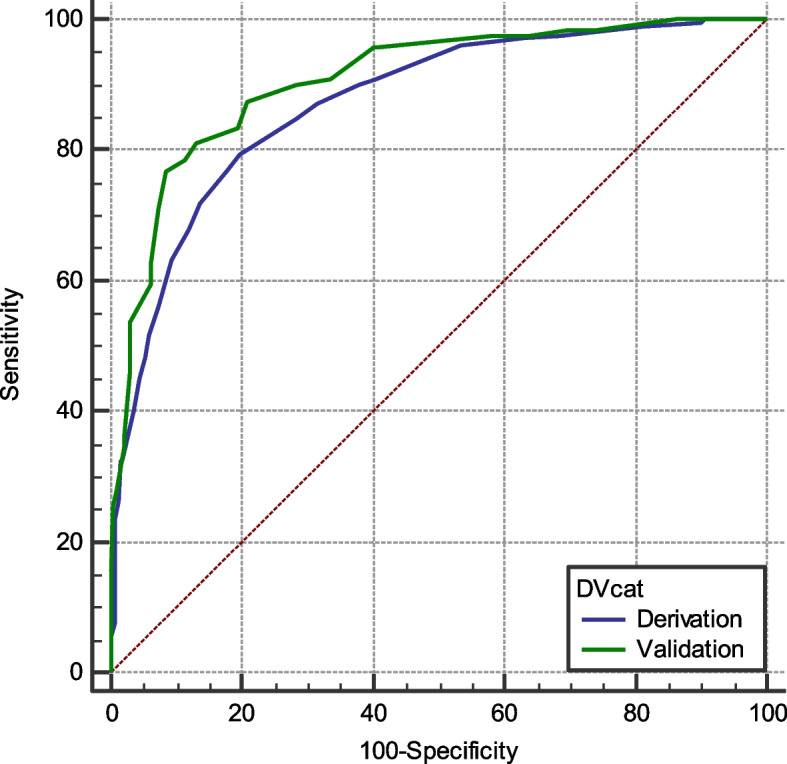
AUROC curves of the derivation and validation cohorts

The accuracy of the novel scoring system (Score A) in the prediction of safe discharge was established by calculating the AUROC of the validation cohort. The AUROC was 0.907 (95% CI 0.870–0.937). This was similar to the AUROC of the derivation cohort (0.872, 95% CI 0.847–0.895; *p* = 0.103) (Fig. 2).

**Fig. 3:**
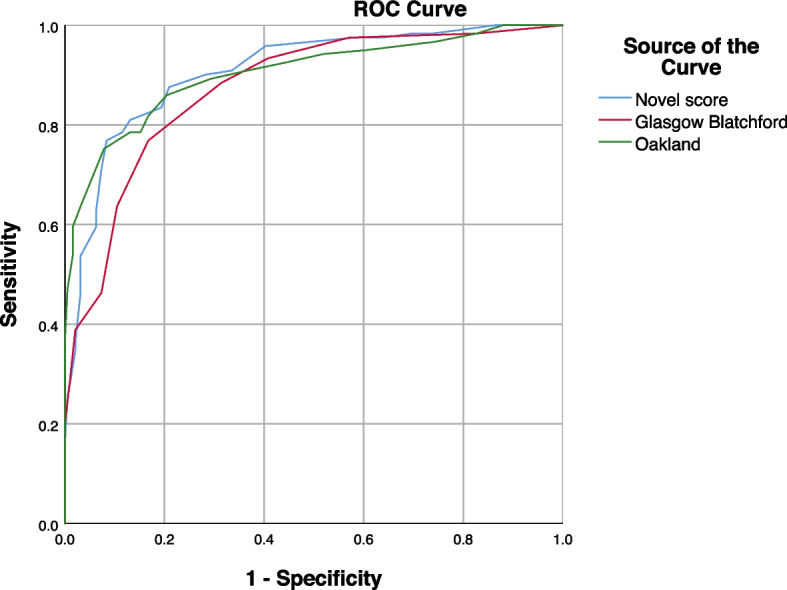
AUROC curves of the novel score and other scoring systems

The AUROC of the novel scoring system was then compared to the AUROCs calculated for the other established scoring systems in the validation cohort (Fig. 3). The AUROCs were then compared as per the method described by DeLong et al. [17] (Table 6). The novel score outperformed the GBS but was otherwise statistically comparable to the Oakland score.

**Table 6:** Comparison of AUROC curves

**Score**	**AUC**	**SE ** ^a^	**95% CI ** ^b^
Novel score	0.907	0.0172	0.870 to 0.937
GlasgowBlatchford	0.874	0.0200	0.832 to 0.909
Oakland	0.903	0.0189	0.865 to 0.934
P-values for comparison of AUROC			
	**Novel score**	GBS	Oakland
**Novel score**	-	**0.0285**	0.7454
GBS	**0.0055**	-	0.0679
Oakland	0.7454	0.0679	-

^a^stands for standard error

^b^stands for 95% confidence interval

The optimal cut-off was determined at 4 points. The cut-off value was determined based on obtaining at least a positive predictive value (PPV) of 95% for safe discharge with the highest Youden’s index when applying the score to the derivation cohort (Table 7). When applied to the validation cohort, a novel score of ≤ 4 points provided a sensitivity of 41.9%, specificity of 97.5%, positive predictive value (PPV) of 96.4% and negative predictive value (NPV) of 51.5% in the prediction of safe discharge for LGITB patients.

**Table 7:** Positive predictive value for safe discharge and Youden’s index for novel score cut-off in the derivation cohort

**Cut-off**	**PPV for safe discharge**	**Youden’s index**
≤ 1	100%	0.094
≤ 1.5	98.0%	0.095
≤ 2	96.9%	0.178
≤ 2.5	95.7%	0.291
≤ 3	95.8%	0.342
≤ 3.5	95.4%	0.383
≤ 4	95.1%	0.425
≤ 4.5	91.7%	0.505

## Discussion

This study has developed and validated a clinical scoring system which can predict for safe discharge in patients admitted via the ED for LGITB. The variables included in our scoring system are previous LGITB admission, Ischemic heart disease history, DRE findings, presence of syncope or dizziness, initial SBP at triage and initial Hb levels. In addition, we have also externally validated other scoring systems which were developed for LGITB, some of which have yet to be validated in an Asian population. Lastly, we have compared our scoring system with others, and concluded that it has a comparable performance with the Oakland score. A score of ≤ 4 has a 96.4% predictive value for safe discharge following admission for LGITB. Lastly, our clinical score can be easily applied by the reviewing clinical team as all the variables would be available following consultation at the ED. A brief history of the patient’s past medical history and current symptoms, a targeted physical examination including a DRE and obtainment of the patient’s vital signs and a full blood count would be all that is required for computation of our clinical score.

Clinical scores can play an important role in the management of patients with UGITB. These scores allow the clinician to accurately identify patients who are either at high risk of adverse clinical events or can be safely managed in the outpatient setting. As the incidence of LGITB increases, there is a pressing need for similar risk stratification scores to be developed for this condition. The 2019 BSG guidelines and 2021 European Society of Gastrointestinal Endoscopy (ESGE) Guidelines have both recommended that a risk assessment tool be incorporated into the initial management of patients presenting with LGITB to guide physician decision on disposition [18]. In recent years, there have been multiple scores developed to predict the clinical outcomes in patients with LGITB. However, these scores have different outcome measures. The Oakland score [16] identified patients who could be safely discharged after presenting with LGITB. The Birmingham score [12] assessed for adverse outcomes (e.g. therapeutic interventions, CT angiography, blood transfusion, 30-day readmission for rebleeding and mortality). The HAKA score [11], STRATE [14], Manraj et al. [15], NOBLADS [13] and SALGIB [7] identified severe LGITB as their primary outcome. In this study, we had chosen the primary endpoint of safe discharge similar to Oakland, as we felt this was the most clinically useful and could optimize the utilization of healthcare resources.

Our score had multiple common variables with the Oakland score: previous LGITB admission, DRE findings, SBP and haemoglobin. However, our score also included 2 new variables: the presence of ischemic heart disease and the presence of syncope or dizziness. We hypothesize that the presence of ischemic heart disease emerged as a significant variable due to the lower blood transfusion thresholds that clinicians may have for this specific population. While the recommended blood transfusion threshold is 7 g/dl for asymptomatic patients, this threshold is increased to 8 g/dl for patients with ischemic heart disease [19]. Hence, patients with ischemic heart disease may be more likely to require transfusion after an admission for LGITB. Dizziness or syncope are symptoms suggestive of anaemia and thus an indicator of the possible need for transfusion. This variable was not evaluated during the derivation of the Oakland score, but had been included as variables in other LGITB risk assessment tools (e.g. NOBLADS, HAKA, STRATE) [11, 13, 14].

While the novel score has a similar AUROC for safe discharge as the Oakland, applicability of the scores in our validation cohort has yielded different outcomes. An Oakland score of ≤ 8 yielded a predictive value of 94.3%, 17.3% sensitivity and 98.3% specificity for safe discharge; however, only 11.2% of the cohort had Oakland scores of ≤ 8. A point cut-off of 4 for our novel scoring system provides a predictive value of 96.4%, with a 41.9% sensitivity and 97.5% specificity for safe discharge. In contrast to the Oakland score (11.2%), 26.7% of the validation cohort fell within the score range of ≤ 4. This indicates that utilization of our score may allow a larger proportion of patients to be safely discharged with similar performance metrics as the Oakland score.

There are several limitations to our study. This is a single tertiary centre study, and thus generalisability to other centres and populations will require further validation. There may also be selection bias as the patients included were those who were admitted via the ED with LGITB. Patients who presented to the ED with LGITB but were subsequently discharged were not included in this study. Hence, the included patient cohort may be biased towards LGITB cases which were more clinically significant with admission deemed necessary. The retrospective nature of the score derivation also meant that variables which were subjective (e.g. patient’s symptoms) may be limited by accuracy of clinical documentation. Nonetheless, we believe the derived novel score will be useful to guide safe discharge after admission for LGITB. Prospective validation efforts are currently on-going and we hope to share the results with the surgical community in the near future.

## Conclusion

In conclusion, we derived and validated a novel scoring system to predict for safe discharge after presentation with LGITB. Prospective validation studies should be performed to assess its applicability in clinical practice.

## Data Availability

The datasets are available from the corresponding author on reasonable request.
